# Nighttime Temperatures and Sunlight Intensities Interact to Influence Anthocyanin Biosynthesis and Photooxidative Sunburn in “Fuji” Apple

**DOI:** 10.3389/fpls.2021.694954

**Published:** 2021-07-23

**Authors:** Xiaomin Xue, Ying Duan, Jinzheng Wang, Fengwang Ma, Pengmin Li

**Affiliations:** ^1^State Key Laboratory of Crop Stress Biology for Arid Areas/Shaanxi Key Laboratory of Apple, College of Horticulture, Northwest A&F University, Xianyang, China; ^2^Shandong Institute of Pomology, Tai’an, China

**Keywords:** low nighttime temperature, light intensity, anthocyanin, photooxidative sunburn, ascorbate

## Abstract

Light and low temperatures induce anthocyanin accumulation, but intense sunlight causes photooxidative sunburn. Nonetheless, there have been few studies of anthocyanin synthesis under different sunlight intensities and low nighttime temperatures. Here, low nighttime temperatures followed by low light intensity were associated with greater anthocyanin accumulation and the expression of anthocyanin biosynthesis genes in “Fuji” apple peel. UDP-glucose flavonoid-3-O-glucosyltransferase (UFGT) activity was positively associated with anthocyanin enrichment. Ascorbic acid can be used as an electron donor of APX to scavenge H_2_O_2_ in plants, which makes it play an important role in oxidative defense. Exogenous ascorbate altered the anthocyanin accumulation and reduced the occurrence of high light–induced photooxidative sunburn by removing hydrogen peroxide from the peel. Overall, low light intensity was beneficial for the accumulation of anthocyanin and did not cause photooxidative sunburn, whereas natural light had the opposite effect on the apple peel at low nighttime temperatures. This study provides an insight into the mechanisms by which low temperatures induce apple coloration and high light intensity causes photooxidative sunburn.

## Introduction

Apples (*Malus domestica* Borkh.) are appreciated by consumers worldwide and provide nutrition in the form of sugars, acids, vitamins, flavonoids, pectin, amino acids, and other components (Boyer and Liu, 2004; Hecke et al., 2006; Zhang et al., 2010). Anthocyanins, members of the flavonoid family, determine the color of the apple and therefore influence its commodity value (An et al., 2020). Anthocyanins also play an important role in plant growth and development (Honda and Moriya, 2018; An et al., 2020), and anthocyanin accumulation enhances the antioxidant capacity of fruit by the removal of reactive oxygen species (Honda and Moriya, 2018).

Anthocyanin biosynthesis is a branch of flavonoid metabolism (Jaakola, 2013; Honda and Moriya, 2018), which includes key enzymes such as chalcone synthase (CHS), dihydroflavonol 4-reductase (DFR), anthocyanidin synthase (ANS), and UDP-glycose flavonoid-3-O-glycosyltransferase (UFGT) (Allan et al., 2008; Jaakola, 2013; Honda and Moriya, 2018). However, anthocyanin biosynthesis begins with 4-coumaroyl CoA from phenylalanine metabolism, and phenylalanine ammonia lyase (PAL) is the key enzyme in phenylalanine metabolism. The genes encoding these enzymes (*PAL*, *CHS*, *DFR*, *ANS*, and *UFGT*) are regulated primarily by the MYB-bHLH-WD40 (MBW) complex (Lloyd et al., 2017; An et al., 2019). In this complex, MYB transcription factors are regarded as the dominant components that regulate the anthocyanin biosynthetic genes (Dubos et al., 2010; Lai et al., 2013). In apple, *MdMYB10* positively regulates the accumulation of anthocyanins by directly affecting the transcript levels of *MdDFR* and *MdUFGT* (Ban et al., 2007; Espley et al., 2007). Other MYB transcription factors such as *MdMYB9*, *MdMYB11*, *MdMYB12*, *MdMYB22*, *MdbHLH3*, and *MdbHLH33* also play important roles in the positive regulation of anthocyanin synthesis (An et al., 2012, 2014; Xie et al., 2012; Wang N. et al., 2018).

Diverse environmental factors affect the synthesis of anthocyanins (Honda and Moriya, 2018; An et al., 2020), and light and temperature have particularly significant effects (Rabino and Mancinelli, 1986). Prolonging the light time or increasing the light intensity was beneficial to the anthocyanin accumulation (Jeong et al., 2004; Lu et al., 2015); the combination of UV-B and UV-C increased the anthocyanin accumulation in green apples (Mabrok et al., 2019). Low temperature induces anthocyanin accumulation by increasing the expression levels of anthocyanin synthesis genes (Xie et al., 2012). *MdbHLH3* promotes anthocyanin synthesis and fruit coloration in response to low temperatures in the apple (Xie et al., 2012). In the red-fleshed apple, low temperatures induce the expression of *MdMYBPA1*, thereby influencing flavonoid biosynthesis (Wang N. et al., 2018; Wang X. et al., 2018). Light intensity is another important factor that affects the anthocyanin content (Rabino and Mancinelli, 1986; Jaakola, 2013), and many studies suggest that light is indispensable for anthocyanin synthesis (Ma and Cheng, 2003; Azuma et al., 2012; Guan et al., 2016). Studies on fruit trees have found that the transcription factors promote the production of anthocyanin by regulating anthocyanin biosynthesis genes in response to UV and blue light (Ubi et al., 2006; Gong et al., 2015; Henry-Kirk et al., 2018; Fang et al., 2019; Ni et al., 2019). Both the wavelength of light and its intensity affect the accumulation of anthocyanin (Jaakola, 2013; Zhang et al., 2018).

Excess light causes photooxidative sunburn characterized by browning, necrosis, and the production of reactive oxygen species (Felicetti and Schrader, 2008). When fruit is moved from a shaded environment to high light conditions, photooxidative damage occurs (Ma and Cheng, 2004). The thermotolerance of sun-exposed apple peel did not differ from that of the shaded peel after different high-temperature treatments under dark conditions (Chen et al., 2008). However, the sun-exposed peel accumulated greater anthocyanin and flavonol contents *via* the phenylpropanoid pathway (Li et al., 2013). In “Fuji” apple peel, photooxidative sunburn causes a reduction in ascorbate (vitamin C) and flavonoid synthesis (Zhang et al., 2015). Ascorbate is a typical antioxidant, and its deficient mutants *vtc1*, *vtc2*, and *vtc3* produce less anthocyanin than wild-type plants (Page et al., 2012; Njus et al., 2020). It is recognized that ascorbate has a major role in scavenging hydrogen peroxide and minimizing photooxidative sunburn (Gatzek et al., 2002; Zhang et al., 2015).

Apple is a kind of fruit tree widely planted in temperate regions of the world. According to the Statistics of the World Food and Agriculture Organization, the cultivation area of apple in the world was 2041.20 thousand hectares and the output was 42.43 million tons in 2019. China is a big apple production country, accounting for 43.27% and 48.63% of the apple cultivation area and yield of the world, both ranking first in the world. In China, “Fuji” apple (*M. domestica* Borkh.) is the most commonly grown major variety and accounts for about 70% of all cultivated apples (Cheng and Zhao, 2019), approximately 90% of which are produced by bagged cultivation (Zhai et al., 2007; Wang et al., 2019). However, bags are removed from “Fuji” apples in October in the major production areas such as the Loess Plateau and the Bohai Bay (Zhang et al., 2016). During this period, daylight is strong and nighttime temperatures are usually low, and it is easy to cause fruit sunburn, which leads to poor coloring, poor appearance quality, and low commodity value. Previous studies focused on the effects of low temperature and light on the anthocyanins synthesis and light on fruit sunburn (Felicetti and Schrader, 2008; Xie et al., 2012; Jaakola, 2013). In this study, the effects of low temperature at night and sunlight intensity at day on the accumulation of anthocyanin and photooxidative sunburn in “Fuji” apple were explored, as well as the regulation of ascorbic acid on anthocyanin metabolism and sunburn, which supplemented the conventional mechanism of low-temperature-promoting anthocyanin metabolism.

## Materials and Methods

### Plant Materials and Treatments

Ten-year-old “Fuji” apple trees (*M. domestica* Borkh.) on M26 rootstocks were used in this study. All trees were planted at a 2.5 m × 3.5 m spacing in an orchard in Qianxian, Shaanxi, China (34.53° N, 108.23° E). They were approximately 4.0 m tall with a central leader, and their crop load was adjusted to 1 fruit/20 cm. The trees were maintained using conventional agricultural practices, including soil, fertilizer, and water management, and disease and pest control measures. Approximately 30 days after full bloom, the fruits were bagged with double-layer paper bags (brown outside and red inside) on May 15, 2014. At about 162 days after full bloom, fruits with bags were picked from the trees on September 24, 2014. When the photoperiod was approximately 12-h light/12-h dark, the fruit bags were then placed under weak light conditions in the laboratory. After the removal of the bag, 50 fruits were selected as the control group, and the remainder was used in the following treatments.

*Experiment 1* (the effect of different nighttime temperatures on fruit sunburn and the effects of daytime light intensity and nighttime temperature on the synthesis and metabolism of anthocyanins and ascorbate): Around 1,470 fruits were divided into three groups and exposed to nighttime temperatures of 5°C, 15°C, and 25°C. The next day before sunrise, samples from these three groups were placed in an open space above four layers of wet gauze and divided into two groups: 70% light intensity (low light) and natural light intensity (high light, the maximum light intensity was around 1,900 ± 20 molμm^–2^ s^–1^ at noon). The light intensity was adjusted by altering the number of mesh and white nylon mesh layers based on solar quantum meter measurements (Technologies Company, IKA Company, Agilent Technology, United States), and full exposure to natural sunlight was considered to represent 100% light intensity. Before sunset, all fruit samples were gathered, their exposed surfaces were marked, and they were returned to their corresponding nighttime temperatures. This procedure was repeated again on the 2nd day and the 3rd day, and samples were obtained at 12, 24, 36, 48, 60, and 72 h. Each treatment was repeated five times with eight fruit per replicate. The level of sunburn on each fruit was assessed based on three categories: no sunburn (SL0), mild sunburn (SL1, the exposed surface of the fruit was white), and severe sunburn (SL2, the exposed surface was brown).

*Experiment 2* (the effects of ascorbate on anthocyanins, hydrogen peroxide, and sunburn under different light intensities): Three hundred and fifty fruits were divided into four groups and soaked in 0, 50, 100, or 150 mM ascorbate overnight (12 h). The next morning before sunrise, the fruits were removed from the soaking solution and placed in an open space above four layers of wet gauze under 70% light intensity (low light) or natural light intensity (high light). The procedure was repeated, and samples were collected at 72 h. Each treatment was repeated five times with eight fruit per replicate.

At each sampling time point, fruit peel disks (1 cm^2^, 1 mm thick) were removed from individual fruits using a peeler and quickly frozen in liquid nitrogen. The frozen samples were mixed in liquid nitrogen, ground into powder using a grinding machine (IKA Company, Germany), and stored at −80°C for further analysis.

### Analysis of Phenolic Compounds

Phenolic compounds were extracted and analyzed as described previously (Zhang et al., 2010). In brief, crushed apple peel (0.5 g) was ground in 1.8 ml of 70% methanol containing 2% formic acid at 0–4°C. The homogenate was centrifuged at 10,000 *g* for 10 min at 4°C. The supernatant was injected into the reaction bottle through a 0.45-μm filter head prior to high-performance liquid chromatography (HPLC) measurement.

Phenolic compounds were analyzed using an Agilent 1200 liquid chromatograph equipped with a diode array detector (Agilent Technology, Palo Alto, CA, United States). Phenolic compounds were separated on an inertsil ODS-3 column (5.0 μm, 4.6 mm × 250 mm) and an ODS-3 guard column (5.0 μm, 4.0 mm × 10 mm). Mobile phase A was a 10% formic acid aqueous solution, and mobile phase B was a 10% formic acid–acetonitrile solution. Gradient elution conditions were 95% A (0 min), 85% A (25 min), 78% A (42 min), 64% A (60 min), and 95% A (65 min). A 10-min run time was performed with a flow rate of 1.0 ml min^–1^ and a column temperature of 30°C. Simultaneous monitoring was performed at 520 nm for cyanidin-3-galactoside and at 365 nm for quercetin-3-glycoside (quercetin-3-*O*-galactoside, quercetin-3-*O*-rutinoside, quercetin-3-*O*-glucoside, quercetin-3-*O*-xyloside, quercetin-3-*O*-arabinoside, and quercetin-3-*O*-rhamnoside). The concentrations of individual phenolic compounds were determined based on peak area and on calibration curves derived from corresponding authentic phenolic compounds; the standards of flavonoids were purchased from Sigma-Aldrich.

### Analysis of Ascorbate Content

The extraction and analysis of ascorbate was performed as described in the study by Li and Cheng (2008). Approximately 0.5 g of sample was mixed with 1.5 ml of 6% perchloric acid in a pre-chilled mortar and ground with a pestle. The crude extract was centrifuged at 12,000 *g* for 20 min at 4°C, and the supernatant was used to measure reduced ascorbate (ASC) and dehydroascorbate (DHA) contents. Thirty microliters of 1.5 M NaCO_3_ was added to 100 μl extract to bring the pH to 1–2. ASC concentration was measured in 200 mM sodium acetate buffer (pH 5.6) before and after a 15-min incubation with 1.5 units of ASC oxidase using a spectrophotometer (265 nm). For total ascorbate, 30 μl of 1.82 M NaCO_3_ was added to 100 μl of extract to raise the pH to 6–7, and the mixture was incubated for 30 min at room temperature with an equal volume (130 μl) of 20 mM reduced glutathione (GSH) in 100 mM Tricine–KOH (pH 8.5). DHA was calculated as the difference between total ascorbate and ASC as described in the study by Zhang et al. (2015).

### Analysis of Anthocyanin-Related Enzyme Activities

Enzymes were extracted as described in the method by Li et al. (2013). For PAL, 1 g of sample was homogenized in 3 ml extraction buffer [100 mM Tris–HCl (pH 8.8), 14 mM β-mercaptoethanol, 5 mM dithiothreitol (DTT), 1% bovine serum albumin (BSA), and 5% polyvinylpolypyrrolidone (PVPP)] at 0–4°C. For DFR, the extraction buffer was the same as PAL, except that the pH was 7.5. For CHS, 2 g of frozen sample was ground in 5 ml extraction buffer [100 mM sodium phosphate buffer (pH 6.8), 14 mM β-mercaptoethanol, 5 mM DTT, 40 mM sodium ascorbate, 3 mM EDTA, 10 μM leupeptin, and 2% BSA] with 5% PVPP at 0–4°C. For ANS, 2 g of sample was homogenized in 5 ml extraction buffer with 5% PVPP [100 mM Tris–HCl buffer (pH 8.0), 14 mM β-mercaptoethanol, 5 mM DTT, 5 mM EDTA, 15 mM MgCl_2_, and 2% BSA]. For UFGT, 0.5 g frozen sample was ground in 1.8 ml of extraction buffer at 0–4°C. The extraction buffer consisted of 100 mM HEPES–KOH (pH 7.5), 10 mM MgCl_2_, 2 mM EDTA, 10 mM DTT, 10% glycerol, 1% BSA, 1% Triton X-100, and 5% insoluble PVPP.

All the above enzyme extracts were desalted by passage through PD10 columns and then used for enzyme analysis immediately. The activities of PAL and DFR were assayed as described in the study by Levis et al. (1998) using a spectrophotometer (UV-2450, Shimadzu, Japan). The absorbance was monitored at 290 nm for PAL and 550 nm for DFR, respectively. For PAL, the reaction system of 1 ml consisted of enzyme extract, 100 mM Tris–HCl buffer (pH 8.8), and 100 mM L-phenylalanine. For DFR, the reaction system of 1 ml consisted of 100 mM Tris–HCl buffer (pH 7.5), 2 mM DTT, enzyme extract, 1 mM glucose-6-phosphate, 1 mM reduced coenzyme II, 5 units of glucose-6-phosphate dehydrogenase, and 15 mM dihydroquercetin. Activities of CHS, ANS, and UFGT were assayed using a 1200 HPLC (Agilent Technology, Palo Alto, CA, United States) as described by Ju et al. (1995) and Pang et al. (2007), respectively. For CHS, the 220 μl reaction system consisted of enzyme extract, 2 mM coumaryl, and 2 mM malonoyl. For ANS, the 500 μl reaction system consisted of 100 mM phosphate buffer (pH 7.0), enzyme extract, 5 M sodium chloride, 0.5 M maltose, 200 mM ascorbic acid, 50 mM ketoglutaric acid, 20 mM ferrous sulfate, and 5 mM EDTA. For UFGT, the 200 μl reaction system consisted of enzyme extract, 3 mM anthocyanidin, 10 mM UDP-galactose, and 200 mM phosphate buffer (pH 7.5).

### Analysis of Ascorbate-Related Enzyme Activities

A UV-Vis spectrophotometer (UV-2450, Shimadzu, Japan) was used to measure the activities of enzymes related to the ascorbate metabolic pathway. Enzymes related to ascorbate conversion were measured as described in the method by Li and Cheng (2008). Monodehydroascorbate reductase (MDHAR) activity was assayed by monitoring the decrease in absorbance at 340 nm because of NADH oxidation in 1-ml assay solution containing 50 mM HEPES–KOH (pH 7.6), 2.5 mM ASC, 0.1 mM NADH, 0.5 unit ASC oxidase, and 20 ml enzyme extract. The reaction was initiated by adding ASC oxidase. Dehydroascorbate reductase (DHAR) activity was determined by monitoring the increase in absorbance at 265 nm because of ASC formation in 1-ml assay solution containing 50 mM HEPES–KOH (pH 7.6), 2.5 mM reduced GSH, 0.1 mM EDTA, 0.2 mM DHA, and 20 ml enzyme extract. The reaction was initiated by adding DHA. Galactose dehydrogenase (GalDH) and galactono lactone dehydrogenase (GalLDH) were measured as described in the study by Gatzek et al. (2002).

### Quantitative Real-Time PCR (qRT–PCR)

RNA isolation and reverse transcription were performed as described previously in the study by Zhang et al. (2003). In brief, the first-strand cDNA was synthesized using the Takara Reverse Transcription Kit, and qRT–PCR was performed using the SYBR premix EX Taq Kit (Takara) to determine the relative expression levels of different genes. Primer Premier 5.0 software was used to design primers for the reference gene *actin* (*MdACT*), *phenylalanine ammonia-lyase* (*MdPAL*), *chalcone synthase* (*MdCHS*), *dihydroflavonol reductase* (*MdDFR*), *anthocyanidin synthase* (*MdANS*), *UDP galactose: flavonoid 3-O-glycosyltransferase* (*MdUFGT*), and *MYB10* (*MdMYB10*), *GDP-L-galactose phosphorylase* (*MdGGP*), *galactose-1-phosphatase* (*MdGPP*), *galactose dehydrogenase* (*MdGalDH*), *galactono lactone dehydrogenase* (*MdGalLDH*), *monodehydroascorbate reductase* (*MdMDHAR*), and *dehydroascorbate reductase* (*MdDHAR*). Three biological replicates were performed for each treatment. All primer sequences are listed in Supplementary Table 1.

### Hydrogen Peroxide Analysis

Approximately 0.5 g of sample was mixed with 1.8 ml of 5% trichloroacetic acid in a mortar, ground with a pestle, and centrifuged at 12,000 *g* for 20 min at 4°C. The supernatant was neutralized with ammonia to pH 7.5, and the hydrogen peroxide content was measured on a spectrophotometer (UV-2450, Shimadzu, Japan) following a previously described method in the study by Chen et al. (2013). The extract was divided into two aliquots of 100 μL. A 20-unit of catalase (CAT) was added to one aliquot (blank). Both the blank and the other aliquot without CAT were added to 0.5 ml with 100 mM Tris–HCl (pH 8.5) and then kept at room temperature for 10 min, following the addition of 0.5 ml colorimetric reagent. The colorimetric reagent was made daily by mixing 1:1 (v/v) 0.3 mM potassium titanium oxalate and 0.3 mM 4-(2-pyridylazo) resorcinol monosodium salt. The assay mixture was incubated at room temperature for 15 min, and then the absorbance at 508 nm was monitored.

### Statistical Analysis

SPSS 16.0 software was used to analyze the statistically significant difference in the data; one-way ANOVA and Duncan’s test were used for analysis (*P* < 0.05).

## Results

### Effect of Low Nighttime Temperature on Anthocyanin Metabolism

#### Anthocyanin and Flavonoid Concentrations

**FIGURE 1:**
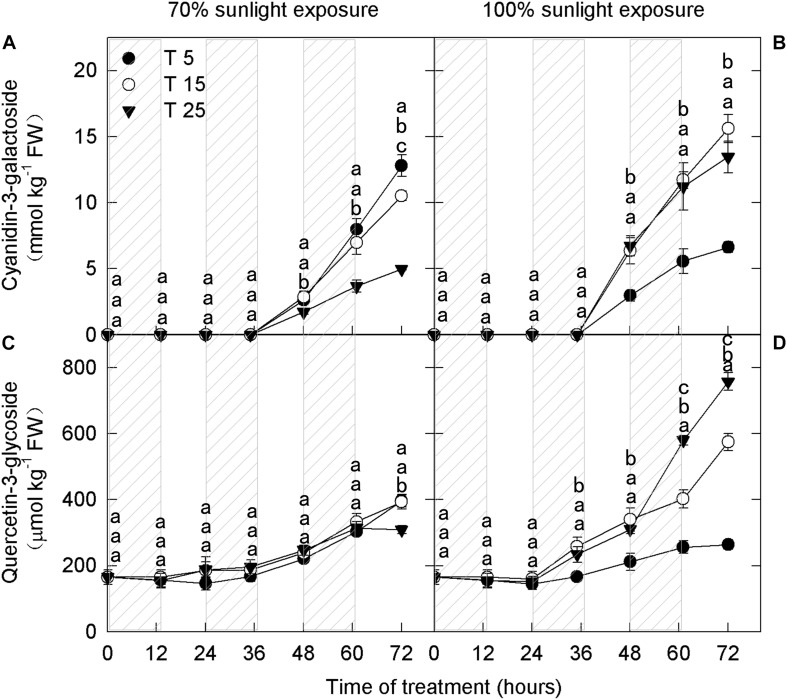
Concentrations of cyanidin-3-galactoside and quercetin-3-glycoside in the peel of “Fuji” apples exposed to different nighttime temperatures and sunlight intensities. Concentrations of cyanidin-3-galactoside in the peel of “Fuji” apples exposed to nighttime temperatures of 5, 15, and 25°C and 70% sunlight intensity **(A)** and 100% sunlight intensity **(B)**. Concentrations of quercetin-3-glycoside in the peel of “Fuji” apples exposed to nighttime temperatures of 5, 15, and 25°C and 70% sunlight intensity **(C)** and 100% sunlight intensity **(D)**. Different lowercase letters indicate significant difference among treatments at P < 0.05.

To explore the influence of low nighttime temperature and sunlight on phenolic compounds, anthocyanins and flavonoids were measured at different time periods. Cyanidin-3-galactoside, which is the most abundant anthocyanin in apple peel, was detected in the samples after 36 h (Figures 1A,B). The concentration of quercetin-3-glycoside, another important flavonoid, increased in apple peel after 24 h (Figures 1C,D).

In fruits exposed to 100% sunlight, cyanidin-3-galactoside levels did not differ between the moderately low nighttime temperature (15°C) treatment and the natural nighttime temperature (25°C) treatment (Figure 1B). However, there was a difference between these treatments in quercetin-3-glycoside concentration after 48 h (Figure 1D). Concentrations of both flavonoid compounds were lower in fruits exposed to low nighttime temperatures (5°C) than in those exposed to 25°C (Figures 1B,D). Because cyanidin-3-galactoside accounts for about 99% of the anthocyanin in apple peel (Chen et al., 2013), the 15°C treatment had no substantive effect on overall anthocyanin content relative to the natural nighttime temperature.

Interestingly, in fruits exposed to 70% sunlight, low nighttime temperatures (5°C and 15°C) caused higher anthocyanin concentrations (Figures 1A,C). In particular, cyanidin-3-galactoside concentration began to increase in the low-temperature treatments after 36 h, and quercetin-3-glycoside concentration rose after 60 h (Figures 1A,C). Overall, anthocyanin concentration decreased as nighttime temperature increased in fruit exposed to 70% sunlight.

#### Transcript Levels of Genes Related to Anthocyanin Synthesis

**FIGURE 2:**
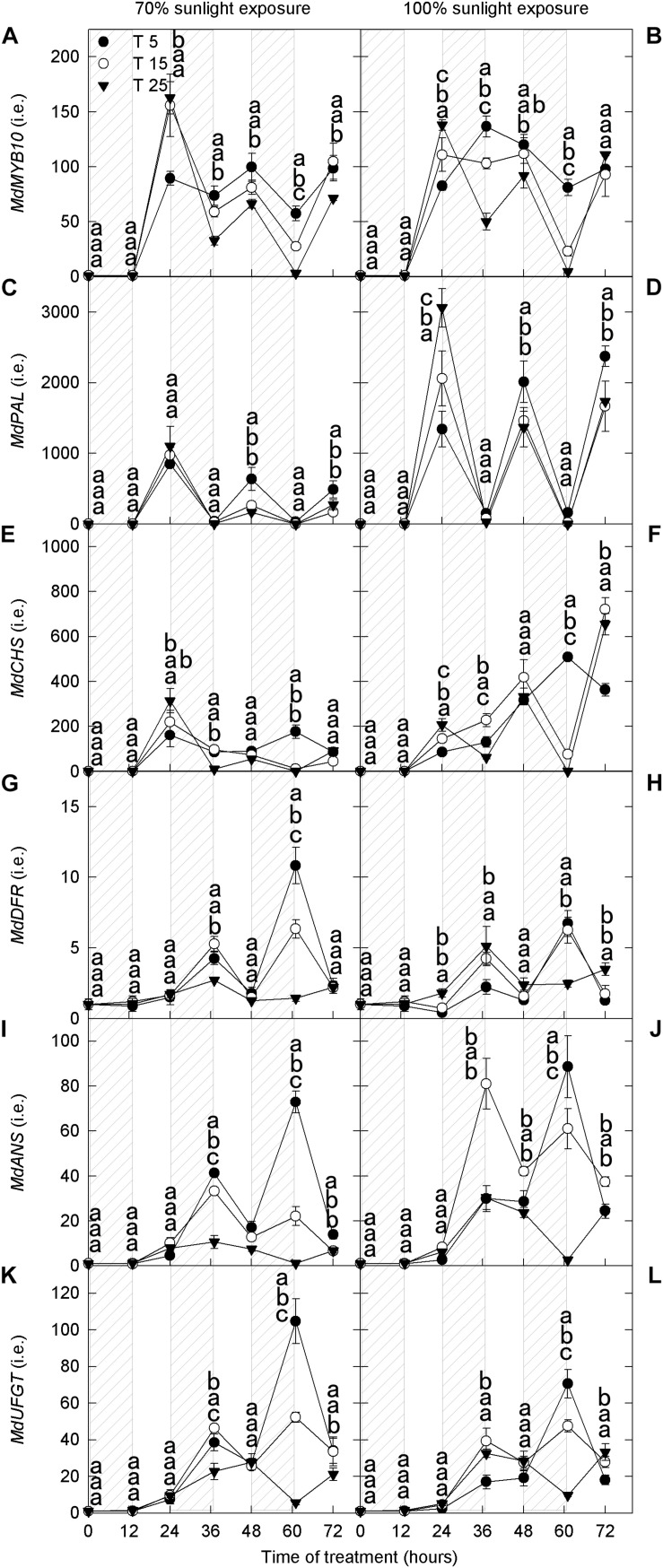
Transcript levels of genes related to anthocyanin synthesis in the peel of “Fuji” apple exposed to different nighttime temperatures and sunlight intensities. The relative transcript level of *MdMYB10* **(A)**, *MdPAL* **(C)**, *MdCHS* **(E)**, *MdDFR* **(G)**, *MdANS* **(I)**, and *MdUFGT* **(K)** in the peel of “Fuji” apples exposed to nighttime temperatures of 5, 15, 25°C and 70% sunlight intensity. The relative transcript level of *MdMYB10* **(B)**, *MdPAL* **(D)**, *MdCHS* **(F)**, *MdDFR* **(H)**, *MdANS* **(J)**, and *MdUFGT* **(L)** in the peel of “Fuji” apples exposed to nighttime temperatures of 5, 15, and 25°C and 100% sunlight intensity. *PAL, phenylalanine ammonia-lyase*; *CHS, chalcone synthase*; *DFR, dihydroflavonol reductase*; *ANS, anthocyanidin synthase*; *UFGT, UDP galactose: flavonoid 3-O-glycosyltransferase*. Different lowercase letters indicate significant difference among treatments at P < 0.05.

Next, we analyzed the expression levels of structural genes (*MdPAL*, *MdCHS*, *MdDFR*, *MdANS*, and *MdUFGT*) and a transcription factor (*MdMYB10*) related to anthocyanin synthesis. There was no marked difference in the expression of *MdMYB10* between the two light intensities (Figures 2A,B), which was similar to that of *MdDFR*, *MdANS*, and *MdUFGT* (Figures 2G–L). However, the expression levels of *MdPAL* and *MdCHS* were lower under 70% light intensity than under natural light (Figures 2C–F). Under both natural light and 70% light intensity at 36 h and 60 h, the expression levels of *MdMYB10* were markedly higher in apple peels exposed to low nighttime temperatures (5°C and 15°C) than in those exposed to 25°C (Figures 2A,B). The expression levels of *MdDFR*, *MdANS*, and *MdUFGT* in the 5°C and 15°C nighttime temperature treatments were high at night and low during the day at 24–72 h under both light intensities, and the expression levels of 5°C were markedly higher than those of 25°C at 36 h and 60 h in 70% light intensity (Figures 2G–L). These results were broadly consistent with the measured concentrations of quercetin-3-glycoside and cyanidin-3-galactoside.

#### Activities of Enzymes Related to Anthocyanin Synthesis

**FIGURE 3:**
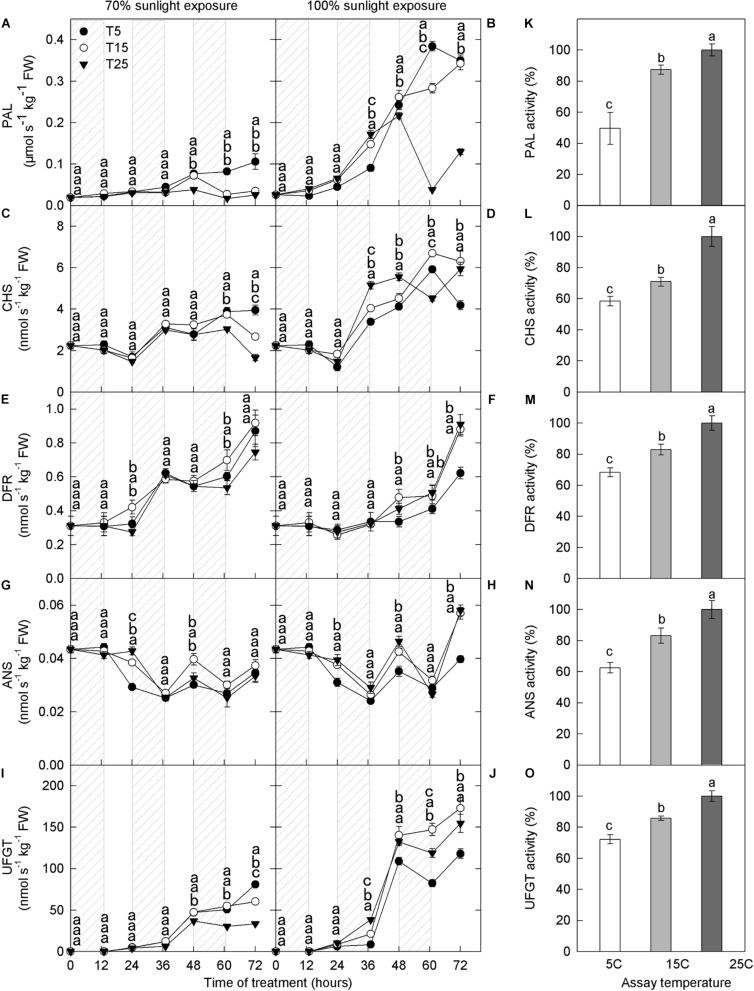
Activities of enzymes in the peel of “Fuji” apple fruits exposed to different nighttime temperatures and sunlight intensities and at different assay temperatures. Enzyme activity of PAL **(A)**, CHS **(C)**, DFR **(E)**, ANS **(G)**, and UFGT **(I)** in the peel of “Fuji” apples exposed to nighttime temperatures of 5, 15, and 25°C and 70% sunlight intensity. Enzyme activity of PAL **(B)**, CHS **(D)**, DFR **(F)**, ANS **(H)**, and UFGT **(J)** in the peel of “Fuji” apples exposed to nighttime temperatures of 5, 15, and 25°C and 100% sunlight intensity. Enzyme activity of PAL **(K)**, CHS **(L)**, DFR **(M)**, ANS **(N)**, and UFGT **(O)** in the peel of “Fuji” apples exposed to assay temperatures of 5, 15, and 25°C. PAL, phenylalanine ammonia-lyase; CHS, chalcone synthase; DFR, dihydroflavonol reductase; ANS, anthocyanidin synthase; UFGT, UDP galactose: flavonoid 3-O-glycosyltransferase. Different lowercase letters indicate significant difference among treatments at P < 0.05.

Enzyme activities mediate anthocyanin synthesis, and we, therefore, measured the activities of enzymes related to anthocyanin. Compared with 70% light intensity, natural light significantly increased the activities of PAL, CHS, and UFGT (Figures 3A–D,I,J). However, DFR and ANS activities were similar under both light intensities (Figures 3E–H). PAL, CHS, DFR, and UFGT activities increased gradually with time, whereas that of ANS showed little change (Figures 3A–J).

Phenylalanine ammonia lyase activity was higher in the low nighttime temperature treatments (5°C and 15°C), and this difference peaked on the 3rd day in both light intensity treatments (Figures 3A,B). Under 100% light intensity, DFR activity was substantially lower in the 5°C treatment at 48–60 h (Figure 3F), and ANS activity was lower in the 5°C treatment at 24–72 h, except 60 h (Figure 3H). UFGT activity increased significantly after 36 h in the low nighttime temperature treatments (5°C and 15°C) at both light intensities (Figures 3I,J). Under 70% light intensity, UFGT activity was significantly higher in the low-temperature treatment than in the 25°C treatment (Figure 3I). However, under natural light, UFGT activity was lower in the 5°C treatment than in the 15°C and 25°C treatments (Figure 3J). Overall, the activities of PAL, CHS, DFR, ANS, and UFGT increased with increasing assay temperature (Figures 3K–O).

### Hydrogen Peroxide Concentration and Photooxidative Sunburn

**FIGURE 4:**
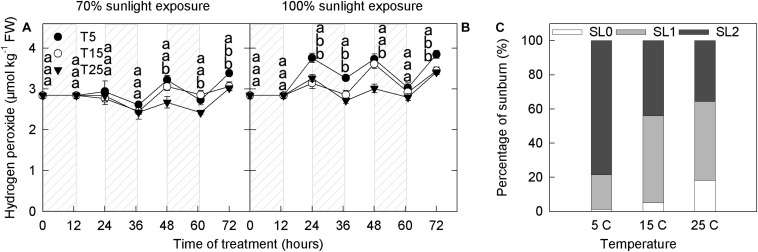
Concentration of hydrogen peroxide in the peel of “Fuji” apple fruits exposed to different nighttime temperatures and sunlight intensities and levels of sunburn under natural light in the peel of fruits exposed to different nighttime temperatures. Concentration of hydrogen peroxide in the peel of “Fuji” apple fruits exposed to nighttime temperatures of 5, 15, and 25°C and 70% sunlight intensity **(A)** and 100% sunlight intensity **(B)**. Levels of sunburn under natural light in the peel of fruits exposed to nighttime temperatures of 5, 15, and 25°C **(C)**. Different lowercase letters indicate significant difference among treatments at P < 0.05.

The anthocyanin concentration increased with the decrease in H_2_O_2_ concentrations, indicating the involvement of anthocyanin in H_2_O_2_ scavenging catalyzed by peroxidase (Vanderauwera et al., 2005), and we, therefore, measured H_2_O_2_ content in apple peels from different treatments. Overall, H_2_O_2_ content increased during the day and decreased at night (Figure 4). Under both 70% light intensity and natural light, H_2_O_2_ content was significantly higher in the samples exposed to 5°C than in those exposed to 25°C at 24–72 h (Figures 4A,B). Under 70% light intensity, peel H_2_O_2_ content began to increase noticeably after the second night of 5°C treatment (Figure 4A). Under natural light, peel H_2_O_2_ content began to increase after one night of 5°C treatment (Figure 4B).

Fruits are sunburned under natural light in all temperature treatments. However, the proportion of severe sunburn was highest in the 5°C treatment (80%), followed by the 15°C treatment (>40%), and was lowest in the 25°C treatment (Figure 4C).

### Effect of Nighttime Temperature on Ascorbate Metabolism

#### Ascorbate Concentration

**FIGURE 5:**
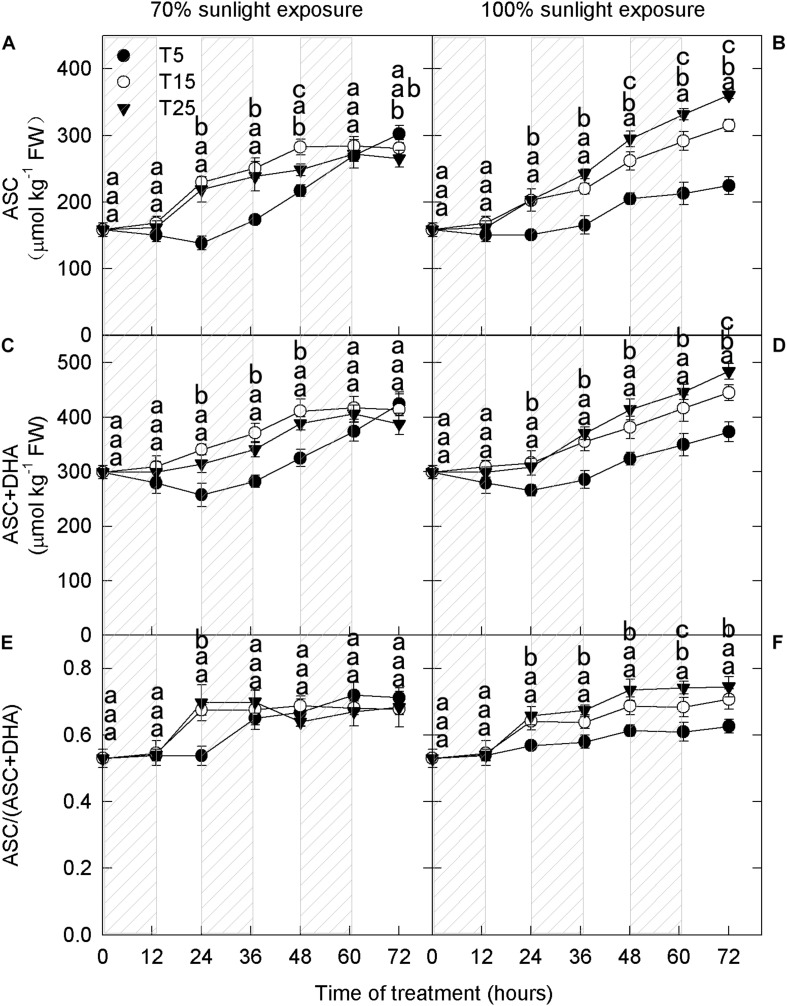
Concentrations of reduced ascorbate and total ascorbate and the ratio of reduced to total ascorbate in the peel of “Fuji” apple fruits exposed to different nighttime temperatures and sunlight intensities. Concentration of reduced ascorbate **(A)**, total ascorbate **(C)**, and the ratio of reduced ascorbate to total ascorbate **(E)** in the peel of “Fuji” apple fruits exposed to nighttime temperatures of 5, 15, and 25°C and 70% sunlight intensity. Concentration of reduced ascorbate **(B)**, total ascorbate **(D)**, and the ratio of reduced ascorbate to total ascorbate **(F)** in the peel of “Fuji” apple fruits exposed to nighttime temperatures of 5, 15, and 25°C and 100% sunlight intensity. Different lowercase letters indicate significant difference among treatments at P < 0.05.

To study the effect of nighttime temperature on the ascorbate metabolism, we measured the concentrations of ASC, total ascorbate (ASC + DHA), and the ratio of reduced ascorbate to total ascorbate [ASC/(ASC + DHA)]. Concentrations of reduced ascorbate and total ascorbate increased gradually with time in the fruit peels (Figures 5A–D), and this increase was significant under natural light (Figures 5B,D).

Under 70% light intensity, the reduced ascorbate concentration was lower in the 5°C treatment than in the 15°C and 25°C treatments on the first 2 days (Figure 5A). However, no significant differences were detected on the 3rd day. Under natural light intensity, the reduced ascorbate concentration was always lower in the 5°C treatment than in the 15°C and 25°C treatments (Figure 5B). Moreover, there were significant differences among different nighttime temperatures. The trends in total ascorbate concentration were the same as those in reduced ascorbate concentration for all temperature and light treatments (Figures 5C,D). Under natural light, the ratio of reduced to total ascorbate was significantly lower in the 5°C treatment (Figure 5F).

#### Transcript Levels of Genes Related to Ascorbate Metabolism

**FIGURE 6:**
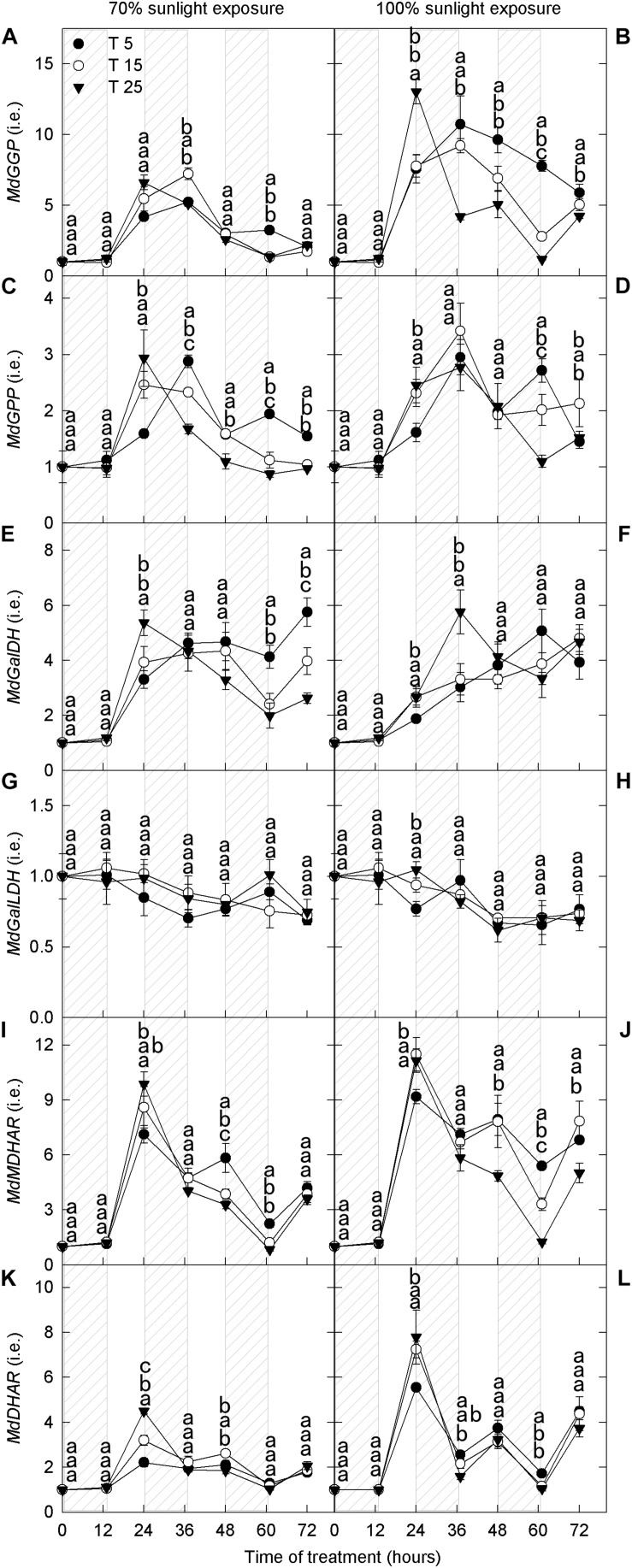
Transcript levels of genes related to ascorbate metabolism in the peel of “Fuji” apple fruits exposed to different nighttime temperatures and sunlight intensities. Relative transcript level of *MdGGP* **(A)**, *MdGPP* **(C)**, *MdGalDH* **(E)**, *MdGalLDH* **(G)**, *MdMDHAR* **(I)**, and *MdDHAR* **(K)** in the peel of “Fuji” apples exposed to nighttime temperatures of 5, 15, and 25°C and 70% sunlight intensity. Relative transcript level of *MdGGP* **(B)**, *MdGPP* **(D)**, *MdGalDH* **(F)**, *MdGalLDH* **(H)**, *MdMDHAR* **(J)**, and *MdDHAR* **(L)** in the peel of “Fuji” apples exposed to nighttime temperatures of 5, 15, and 25°C and 100% sunlight intensity. *GGP, GDP-L-galactose phosphorylase*; *GPP, galactose-1-phosphatase*; *GalDH, galactose dehydrogenase*; *GalLDH, galactono lactone dehydrogenase*; *MDHAR, monodehydroascorbate reductase*; and *DHAR, dehydroascorbate reductase*. Different lowercase letters indicate significant difference among treatments at P < 0.05.

GDP-L-galactose phosphorylase (GGP), galactose-1-phosphatase (GPP), GalDH, and GalLDH are the four enzymes involved in ascorbate synthesis. MDHAR and DHAR are the two enzymes that convert oxidized ascorbate to reduced ascorbate. Under 70% light intensity, the expression levels of MdGGP, MdGalLDH, and MdDHAR did not differ among nighttime temperature treatments (Figures 6A,G,K). The expression levels of *MdGPP*, *MdGalDH*, and *MdMDHAR* were low for the first 12 h and then increased rapidly; their expression levels were markedly higher in the 5°C treatment than in the 25°C treatment after 36 h (Figures 6C,E,I). Under natural light, the expression levels of *MdGGP*, *MdGPP*, *MdGalDH*, *MdMDHAR*, and *MdDHAR* were low for the first 12 h and then increased (Figures 6B,D,F,J,L). However, the expression levels of *MdGPP*, *MdGalDH*, *MdGalLDH*, and *MdDHAR* did not differ among nighttime temperature treatments (Figures 6D,F,H,L). The expression levels of *MdGGP* and *MdMDHAR* were higher in the 5°C treatment after 36 h than in the 25°C treatment (Figures 6B,J).

#### Activities of Enzymes Related to Ascorbate Metabolism

**FIGURE 7:**
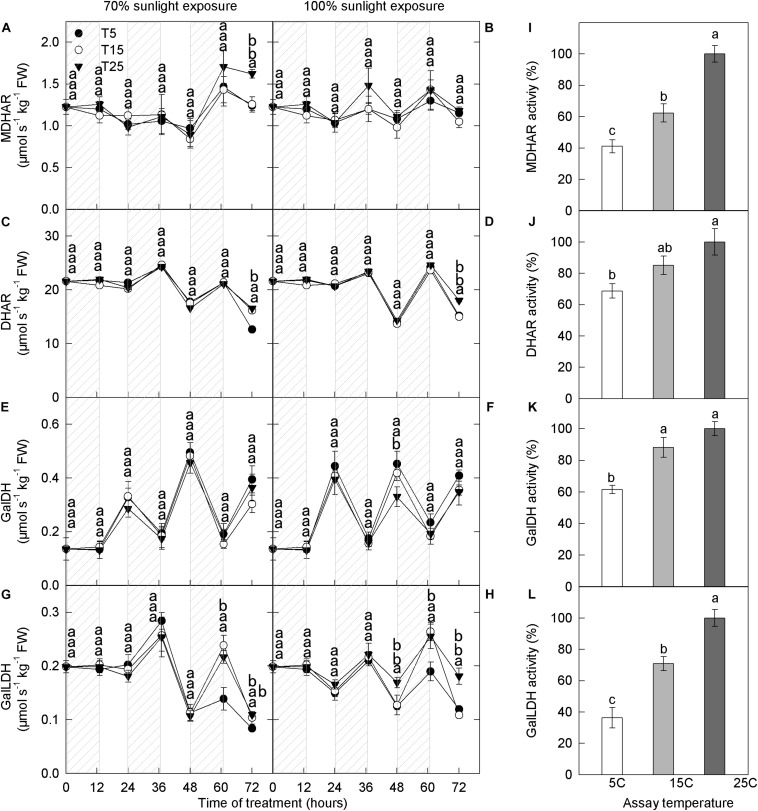
Activities of enzymes related to ascorbic acid metabolism in the peel of “Fuji” apple fruits exposed to different nighttime temperatures and sunlight intensities and measured at different assay temperatures. Enzyme activity of MDHAR **(A)**, DHAR **(C)**, GalDH **(E)**, and GalLDH **(G)** in the peel of “Fuji” apples exposed to nighttime temperatures of 5, 15, and 25°C and 70% sunlight intensity. Enzyme activity of MDHAR **(B)**, DHAR **(D)**, GalDH **(F)**, and GalLDH **(H)** in the peel of “Fuji” apples exposed to nighttime temperatures of 5, 15, and 25°C and 100% sunlight intensity. Enzyme activity of MDHAR **(I)**, DHAR **(J)**, GalDH **(K)**, and GalLDH **(L)** in the peel of “Fuji” apples exposed to assay temperatures of 5, 15, and 25°C. MDHAR, monodehydroascorbate reductase; and DHAR, dehydroascorbate reductase; GalDH, galactose dehydrogenase; GalLDH, galactono lactone dehydrogenase. Different lowercase letters indicate significant difference among treatments at P < 0.05.

We next measured the activities of enzymes related to ascorbate metabolism. At both light intensities, MDHAR activity remained relatively stable through time, whereas DHAR activity decreased slightly (Figures 7A–D). No significant differences in MDHAR and DHAR activities were detected among the different nighttime temperature treatments. The activity of GalDH was high during the day and low at night, but there were no differences in GalDH activity among the different nighttime temperature treatments (Figures 7E,F). GalLDH activity was low during the day and high at night, opposite to that of GalDH, and there was no effect of nighttime temperature on GalLDH activity (Figures 7G,H). MDHAR, DHAR, GalDH, and GalLDH activities increased with assay temperature: The lowest and highest activities were recorded at 5°C and 25°C, respectively (Figures 7I–L).

### Effect of Exogenous Ascorbate on Anthocyanin Metabolism

#### Anthocyanin and Flavonoid Concentrations

**FIGURE 8:**
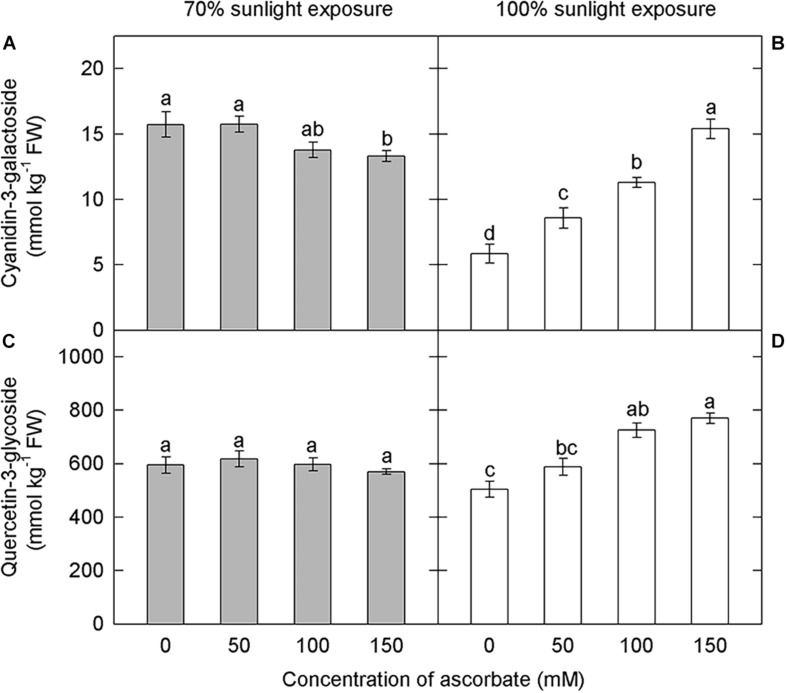
Effect of exogenous ascorbate on the concentration of cyanidin-3-galactoside and quercetin-3-glycoside in the peel of “Fuji” apple fruits. Concentrations of cyanidin-3-galactoside **(A)**, quercetin-3-glycoside **(C)** in the peel of “Fuji” apples soaked in ascorbic acid of 0, 50, 100, and 150 mM and 70% sunlight intensity. Concentrations of cyanidin-3-galactoside **(B)**, quercetin-3-glycoside **(D)** in the peel of “Fuji” apples soaked in ascorbic acid of 0, 50, 100, and 150 mM and 100% sunlight intensity.

Since ascorbate has a major role in hydrogen peroxide scavenging and the avoidance of photooxidative sunburn, we investigated the influence of ascorbate on anthocyanin and flavonoid concentrations. Under 70% light intensity, cyanidin-3-galactoside concentrations were significantly higher under lower concentrations of exogenous ascorbic acid (0 and 50 mM) than under the highest concentration (150 mM) (Figure 8A). However, exogenous ascorbate had no effect on quercetin-3-glycoside concentrations at this lower light intensity (Figure 8C). By contrast, cyanidin-3-galactoside and quercetin-3-glycoside concentrations increased significantly and linearly with exogenous ascorbate concentration under natural light conditions (Figures 8B,D). Anthocyanins in samples exposed to 70% light intensity were less affected by exogenous ascorbate concentration than those in samples exposed to natural light.

#### Hydrogen Peroxide Concentration

**FIGURE 9:**
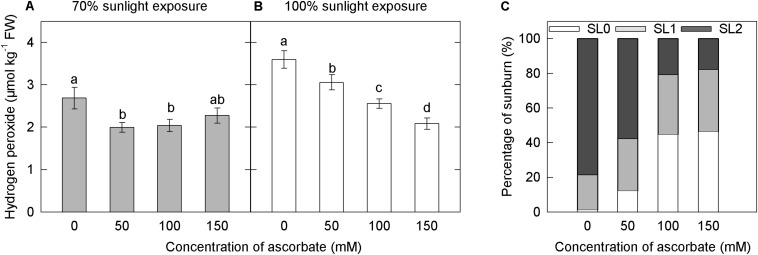
Effects of exogenous ascorbate on the concentration of hydrogen peroxide and the percentage of sunburn under natural light conditions in the peel of “Fuji” apple fruits. Concentration of hydrogen peroxide in the peel of “Fuji” apple fruits soaked in ascorbate of 0, 50, 100, and 150 mM and 70% sunlight intensity **(A)** and 100% sunlight intensity **(B)**. The percentage of sunburn in the peel of “Fuji” apple fruits soaked in ascorbic acid of 0, 50, 100, and 150 mM under natural light conditions **(C)**.

Under both light intensities, H_2_O_2_ concentration was significantly higher in the untreated samples than in samples treated with 50, 100, and 150 mM ascorbate (Figures 9A,B). Overall, exogenous ascorbate reduced the concentration of H_2_O_2_ in apple peel. In the 70% light intensity treatment, there were no significant differences in H_2_O_2_ concentration among samples treated with different concentrations of exogenous ascorbate (Figure 9A). In the natural light treatment, however, H_2_O_2_ concentration decreased as exogenous ascorbate concentration increased (Figure 9B). Moreover, the overall H_2_O_2_ concentration was significantly lower under 70% light intensity than that under natural light intensity (Figures 9A,B). This result suggested that the shading treatment significantly reduced H_2_O_2_ production in the peel.

#### Photooxidative Sunburn

Under 70% light intensity, no sunburn occurred in fruits treated with different concentrations of ascorbate (data not shown). However, under natural light, the percentage of severe sunburn decreased as the concentration of exogenous ascorbate increased (Figure 9C). The application of ascorbate appeared to reduce the level of photooxidative sunburn caused by strong light.

## Discussion

Previous studies indicate that low temperature causes the accumulation of anthocyanins (Shvarts et al., 1997; Ubi et al., 2006; Fang et al., 2019; Gao-Takai et al., 2019), due to the adaptation strategy to the low temperatures (Zhang et al., 2019). Considering the range of possible nighttime temperatures, it is particularly important to explore the influence of nighttime low temperatures on anthocyanin accumulation. In this study, we revealed a close association between the effects of low nighttime temperature and light intensity on anthocyanin accumulation. Under 70% light intensity during the day, anthocyanin concentration in apple peels was significantly higher in the 5°C and 15°C nighttime temperature treatments than in the 25°C nighttime treatment (Figure 1A), consistent with previous studies (Shvarts et al., 1997; Ubi et al., 2006; Fang et al., 2019; Gao-Takai et al., 2019). However, under natural light, anthocyanin concentrations were lowest in the 5°C nighttime temperature treatment (Figure 1B). These results showed that the effect of low nighttime temperature on anthocyanin accumulation in “Fuji” apple peel was strongly influenced by daytime light intensity.

It is recognized that low temperature promotes the expression of anthocyanin synthesis-related genes (Ban et al., 2007; Xie et al., 2012; Tian et al., 2015). Under both light treatments, the expression levels of structural genes (*MdCHS*, *MdDFR*, *MdANS*, and *MdUFGT*) and one transcription factor gene (*MdMYB10*) related to anthocyanin synthesis were higher in the low nighttime temperature treatment mostly (Figure 2). However, under natural light, the activities of CHS, DFR, ANS, and UFGT did not increase after low nighttime temperature treatment (Figure 3). This result may reflect posttranscriptional protein modifications, which take place during the process of mRNA translation and subsequent folding of the enzyme protein. It should be noticed that the enzyme activity might include that of the isoenzymes, and it might be not reflected by the expression levels of a single gene. Further studies are needed to clarify this. UFGT is the most critical enzyme in the anthocyanin biosynthetic pathway. In the 70% light intensity treatment, the activity of UFGT was higher in the 5°C and 15°C nighttime treatments than in the 25°C nighttime treatment. Interestingly, in the natural light treatment, UFGT activity was lower in the 5°C treatment than in the 15°C and 25°C treatments (Figures 3I,J). The trend in UFGT activity was consistent with that of anthocyanin concentration, and UFGT activity may have therefore directly influenced anthocyanin synthesis in “Fuji” apple. Moreover, the influence of different nighttime temperatures on anthocyanin synthesis in apple peel mainly occurred at the protein level.

Photooxidative sunburn is also referred to as light stress–induced sunburn (Chen et al., 2008; Zhang et al., 2015; Jia et al., 2019). With the extension of the experimental period, apple peel under natural light intensity experienced different degrees of sunburn. Apples from the 5°C treatment group had the most severe sunburn, followed by those from the 15°C and 25°C groups (Figure 4). However, no sunburn was observed under 70% light intensity. These findings indicate that sunburn was caused by the interaction between low nighttime temperature and strong daytime sunlight. Zhang et al. (2015) have previously shown that the severity of sunburn in fruit peel is linked to hydrogen peroxide accumulation. Here, hydrogen peroxide concentration was higher in the 5°C nighttime treatment than in the 15°C and 25°C nighttime treatments from the 1st day, particularly under natural light (Figure 4). Moreover, PAL activity was relatively high in the samples under natural light (Figure 3). When exposed to excessive natural light, PAL catalyzes the production of various quinines that cause peel browning (Ke and Saltveit, 1989; Peiser et al., 1998). Under natural light, the ascorbate concentration was significantly lower in fruits from the 5°C treatment than in those from the 15°C and 25°C treatments from the 1st day (Figure 5). Moreover, quercetin-3-glycoside content was lower from the 2nd day (Figure 1). Because flavonoids can absorb ultraviolet and visible light, a reduction in their concentration would expose the fruit to relatively higher light conditions, resulting in the formation of more reactive oxygen species in the peel (Harborne and Williams, 2000; Ferreyra et al., 2012). Higher ROS production and lower ROS scavenging capacity in fruit exposed to a 5°C nighttime temperature and natural daylight conditions, therefore, led to sunburn.

Ascorbate removes ROS from plants through several pathways and thereby plays a significant role in the prevention of photooxidative stress (Li and Schellhorn, 2007; Smirnoff, 2018; Njus et al., 2020). Page et al. (2012) showed that ascorbate affected the anthocyanin content through a decline in the activity of enzymes and the expression of structural genes related to the anthocyanin biosynthetic pathway in *Arabidopsis*. In the present study, the ascorbate concentration was the lowest in samples exposed to 5°C (Figure 5). However, under 70% light intensity, the anthocyanin concentration was significantly higher in fruits from the 5°C nighttime temperature treatment (Figure 1). Based on these opposite trends, we infer that ascorbate did not affect anthocyanin accumulation through the direct regulation of anthocyanin synthesis.

Furthermore, the lower concentrations of exogenous ascorbate (50, 100 mM) did not influence the accumulation of anthocyanins in the apple peel under 70% light intensity. Under natural light, anthocyanin concentrations increased linearly with the concentration of exogenous ascorbate (Figure 8). In other words, the effect of ascorbate on anthocyanin accumulation in the apple peel was closely related to external light intensity. In addition, H_2_O_2_ content was significantly lower under 70% light intensity than under natural light (Duncan’s test was used for the statistical analysis), and H_2_O_2_ content decreased with increasing exogenous ascorbate concentration under natural light (Figure 9). Under natural light, ascorbate concentration in fruits was significantly lower in the 5°C treatment group, and ROS cleared by APX were reduced. Ascorbate, a fundamental antioxidant, acts as a substrate in the xanthophyll cycle that converts violaxanthin to antheraxanthin and zeaxanthin. Therefore, the rate of ROS production was greater than the rate of ROS elimination. As a result, a large amount of ROS was accumulated and fruits became sunburned, thereby affecting the anthocyanin synthesis and ultimately reducing the anthocyanin accumulation. However, under 70% light intensity, despite low ascorbate concentration and high hydrogen peroxide concentration in fruits treated at 5°C, the production and removal of ROS remained in a dynamic balance. Consequently, no sunburn occurred in fruits and anthocyanins were accumulated. Therefore, ascorbate appears to influence the anthocyanin accumulation in apples primarily through the removal of ROS produced in the peel.

## Conclusion

The effect of low nighttime temperature on anthocyanin accumulation in the peel of “Fuji” apple was closely related to sunlight intensity. Under low sunlight intensity, fruits had no sunburn, and low nighttime temperature treatment was beneficial for the accumulation of anthocyanin. At higher sunlight intensity, anthocyanin concentration in fruits decreased significantly in the low nighttime temperature treatment. The activity of UFGT played a significant role in regulating the effect of low nighttime temperature on the anthocyanin content in the fruit peel. In addition, the effect of ascorbate on the anthocyanin content in the peel of “Fuji” apple was related to sunlight intensity. Under natural daylight, the fruits had sunburn after low nighttime temperature treatment because of the decline in ascorbate content and the accumulation of reactive oxygen species. As a result, the content of anthocyanins in the apple peel decreased.

## Data Availability Statement

The original contributions presented in the study are included in the article/Supplementary Material, further inquiries can be directed to the corresponding author.

## Author Contributions

PL, XX, and YD designed the experiment. YD and XX performed the experiment and analyzed the data. XX and PL wrote the manuscript. FM and JW read and approved the manuscript. All authors contributed to the article and approved the submitted version.

## Conflict of Interest

The authors declare that the research was conducted in the absence of any commercial or financial relationships that could be construed as a potential conflict of interest.

## Publisher’s Note

All claims expressed in this article are solely those of the authors and do not necessarily represent those of their affiliated organizations, or those of the publisher, the editors and the reviewers. Any product that may be evaluated in this article, or claim that may be made by its manufacturer, is not guaranteed or endorsed by the publisher.

## Supplementary Material

Supplementary Table 1 Primer sequences used in the study.
