# Supplementary material for: Nighttime Temperatures and Sunlight Intensities Interact to Influence Anthocyanin Biosynthesis and Photooxidative Sunburn in “Fuji” Apple
Source: Front Plant Sci. 2021 Jul 23;12:694954. doi: 10.3389/fpls.2021.694954 (PMC8343144; doi:10.3389/fpls.2021.694954)
Supplement: Supplementary Table 1 — Primer sequences used in the study. [file Table_1.DOC]

**Supplemental Table S1: Primer sequences used in the study**

| Gene name | Forward primer | Reverse primer |
| --- | --- | --- |
| *MdActin* | TGACCGAATGAGCAAGGAAATTACT | TACTCAGCTTTGGCAATCCACATC |
| *MdPAL* | TTGACGCACAAGTTGAAGCA | CCACTGAGGTGATGTTCGGA |
| *MdCHS* | GGAGACAACTGGAGAAGGACTGGAA | CGACATTGATACTGGTGTCTTCA |
| *MdDFR* | GATAGGGTTTGAGTTCAAGTA | TCTCCTCAGCAGCCTCAGTTTTCT |
| *MdANS* | CCAAGTGAAGCGGGTTGTGCT | CAAAGCAGGCGGACAGGAGTAGC |
| *MdUFGT* | TCTACGCCCGAGGGATGTT | ACCGTTTTGAGGTCTCCTGTTT |
| *MdMYB10* | TGCCTGGACTCGAGAGGAAGACA | CCTGTTTCCCAAAAGCCTGTGAA |
| *MdGGP* | CAGAAAGATGAGGCGGAGGA | GAAGTTTTGCCCCTGGAATG |
| *MdGPP* | GATTCTCTTGCTCAGTTCCTCTC | TGCTCCACATGCTTGGTCTC |
| *MdGalDH* | GCCGAGGCATCAACTTCTTT | CGTCAGCATAGCGTCCACA |
| *MdGalLDH* | ATGCCCACGAGAAGAAATCC | TCCTTATCCACCTCCAAAACCT |
| *MdMDHAR* | CCATACTTCTATTCCCGCTCCT | CGACCACCTTCCCGTCTTT |
| *MdDHAR* | AGTGGACGGTTCCAGCAGA | TTCCCATCCCGCAATCAC |
